# RNAi pathways repress reprogramming of *C. elegans* germ cells during heat stress

**DOI:** 10.1093/nar/gkaa174

**Published:** 2020-03-18

**Authors:** Alicia K Rogers, Carolyn M Phillips

**Affiliations:** Department of Biological Sciences, University of Southern California, Los Angeles, CA 90089, USA

## Abstract

Repression of cellular reprogramming in germ cells is critical to maintaining cell fate and fertility. When germ cells mis-express somatic genes they can be directly converted into other cell types, resulting in loss of totipotency and reproductive potential. Identifying the molecular mechanisms that coordinate these cell fate decisions is an active area of investigation. Here we show that RNAi pathways play a key role in maintaining germline gene expression and totipotency after heat stress. By examining transcriptional changes that occur in *mut-16* mutants, lacking a key protein in the RNAi pathway, at elevated temperature we found that genes normally expressed in the soma are mis-expressed in germ cells. Furthermore, these genes displayed increased chromatin accessibility in the germlines of *mut-16* mutants at elevated temperature. These findings indicate that the RNAi pathway plays a key role in preventing aberrant expression of somatic genes in the germline during heat stress. This regulation occurs in part through the maintenance of germline chromatin, likely acting through the nuclear RNAi pathway. Identification of new pathways governing germ cell reprogramming is critical to understanding how cells maintain proper gene expression and may provide key insights into how cell identity is lost in some germ cell tumors.

## INTRODUCTION

Germ cells must repress expression of somatic genes and repetitive elements in order to maintain totipotency and reproductive potential from one generation to the next. When this gene repression fails, germ cells can prematurely differentiate into other cell types, similar to germline teratomas, which are tumors formed from either male or female germ cells that contain various somatic tissues including teeth, hair, muscle, and bone. In *C. elegans*, germ cell differentiation into somatic cells, such as muscle or neurons, has been observed following disruption of transcriptional regulators, such as histone modifying enzymes, translational regulators or P granules (1–8). P granules, which are perinuclear RNA–protein granules, contain many known regulators of translation and are thought to act as a hub for post-transcriptional regulation (9). Interestingly, this cellular differentiation upon P granule disruption is not accompanied by up-regulation of somatic transcripts in day one adults (10), but is associated with increased somatic transcripts in older animals (11).

RNA silencing, or RNA interference (RNAi), is an evolutionarily conserved pathway that is employed to regulate endogenous and exogenous gene expression. Integral components of RNAi pathways are RNA-induced silencing complexes (RISC)—made up of Argonaute protein family members and their associated small RNAs—which transcriptionally and post-transcriptionally regulate target transcripts (12–18). The small RNA guides can be generated through several mechanisms that generate small RNAs with distinct features. Small-interfering RNAs (siRNAs) can be generated from exogenous and endogenous long double-stranded RNAs (dsRNAs) cleaved by the RNase III-like enzyme Dicer (19,20). In *C. elegans*, these siRNAs are referred to as primary siRNAs because they initiate amplification of additional secondary siRNAs (21–25). RNA-dependent RNA polymerases (RdRPs) participate in this amplification step within a perinuclear granule referred to as the *Mutator* focus and associate with the *mutator* complex proteins (21,23–26). Secondary siRNAs dependent on the *mutator* complex are synthesized in an antisense orientation relative to mRNA target transcripts and are essential for robust RISC-mediated silencing (21–25,27,28). Classical RNAi post-transcriptionally regulates targets in the cytoplasm; however RNAi can also occur in the nucleus – termed nuclear RNAi—to transcriptionally regulate mRNAs at the chromatin level by directing the establishment of the repressive chromatin mark H3K9me3 at target loci (12,13,16,17,29). Classical and nuclear RNAi occurs in both somatic cells and germ cells (29,30). In the germ cells, many of the proteins associated with RNAi, including multiple Argonaute proteins, localize to the P granule and adjacent to nuclear pore complexes (22,31–35). RISC-meditated regulation is responsible for maintaining homeostasis, appropriate gene expression, silencing of foreign genetic elements, and ultimately plays key roles in development and fertility (14,36).

While mutants for components of the *mutator* complex are viable, their fertility is more sensitive to environmental conditions, such as heat stress, compared to the fertility of wild-type animals. In general, *mutator* complex mutants have increased incidence in male progeny, as a direct result of chromosome nondisjunction events, and increased transposition of transposable elements (TEs) in their germline genome (37,38). In addition, many mutants in small RNA pathway genes exhibit a progressive generational decrease in brood size towards sterility—termed the Mortal Germline (Mrt) phenotype. For example, mutations in the *C. elegans* ortholog of Piwi, *prg-1*, which associates with piRNAs, become sterile after approximately 25 generations of homozygosity, regardless of temperature (39). Other germline RNAi mutants only exhibit a Mrt phenotype when cultured in specific stressful conditions: mutants in the nuclear RNAi pathway genes, *nrde-2* and *hrde-1*, become sterile after 3–5 generations at elevated temperature while mutants of the RNAi factor *rsd-2* require 8–12 generations at elevated temperature to become sterile (12,40). Thus, depending on the RNAi factor mutated, the rate of the heat-sensitive Mrt phenotype, and ultimately the generation at which sterility occurs, differs greatly (12,37,40–43). In contrast to the Mrt phenotypes observed in *rsd-2* and the nuclear RNAi pathway mutants, a null mutation in *mut-16*, an essential component of the *mutator* small RNA amplification pathway and the nucleating factor of the perinuclear *Mutator* foci, causes heat-sensitive sterility after a single generation when the mutants are cultured at elevated temperature (26,37,44). The temperature-sensitive sterility observed in *mutator* complex mutants implicates this pathway in playing an important role in maintaining the integrity and fitness of the germline from one generation to the next under heat stress. However, how heat stress triggers transgenerational sterility, and what determines the rate of progressive decline in fertility, in *mutator* complex pathway mutants remains unknown.

Epigenetic regulators also play key roles in the maintenance of germline fertility. For example, mutations in the H3K4 methyltransferase, *set-2*, have a progressive loss of fertility and produce increasing numbers of inviable embryos when cultured at elevated temperature for several generations (45,46). The germlines of these animals display endomitotic oocytes indicating a premature entry into the cell cycle, masculinized germlines where the normal switch to oogenesis has failed, or otherwise disorganized nuclei with abnormal cellular morphology (45). Similarly, mutations in the H3K9 methyltransferase, *met-2*, or the H3K4me2 demethylase, *spr-5*, also result in transgenerational sterility phenotypes (47–51). Furthermore, disruption of the H3K27 methylation status within germ cells permits conversion of the germ cells into different somatic cell lineages (3,6,7). This similarity in fertility defects between mutants of the chromatin and RNAi pathways suggests that the intersection of these pathways may play a role in the maintenance of fertility and totipotency, particularly during heat stress.

Here, we demonstrate that upon heat stress, MUT-16 and the *mutator* small RNA amplification pathway are required to maintain germline gene expression patterns. By performing comparative bioinformatic analysis on transcriptional changes in *mut-16* and control animals cultured at permissive and elevated temperature, we observed aberrant expression of somatic genes only when *mut-16* mutants were exposed to heat stress. *hrde-1* mutants cultured for multiple generations at elevated temperature also exhibit increased expression of somatic genes that correlate with their heat stress-induced Mrt phenotype. Interestingly, small RNAs targeting the mis-expressed somatic genes were not significantly affected in heat stressed *mut-16* mutants compared to controls. This result indicates that loss of small RNAs targeting the mis-expressed somatic genes is unlikely to be the sole cause of their increased expression. We also observed a modest increase in the expression of tandem and inverted repeats in heat-stressed *mut-16* mutants. These repetitive elements are direct targets of the *mutator* small RNA amplification pathway; however, their mRNA expression is only elevated after loss of the targeting small RNAs in combination with heat stress. Loss of P granules is associated with similar dis-regulation of somatic genes, and indeed, we observed disruption of P granule localization in *mut-16* mutants at elevated temperature. However, P granule loss is unlikely to be the sole trigger of the somatic gene expression in the germline of RNAi pathway mutants because the transcriptional changes in heat-stressed RNAi pathway mutants occur earlier than the transcriptional changes in P granule knockdown animals. Furthermore, we see increased chromatin accessibility along the gene bodies of the somatic genes up-regulated upon heat stress in *mut-16* mutants. Thus, our data indicates that the *mutator* small RNA amplification pathway is critical to maintain germline totipotency during heat stress, likely acting in part through the nuclear RNAi pathway and the regulation of germline chromatin.

## MATERIALS AND METHODS

### *C. elegans* strains

Unless otherwise stated, worms were grown at 20°C according to standard conditions (52). All strains are in the N2 background. Strains used include:

N2 – wild-type.
NL1810 – *mut-16(pk710) I*.
DUP75 – *pgl-1(sam33[pgl-1::GFP::3xFLAG]) IV*.
USC1252 – *mut-16(pk710) I; pgl-1(sam33[pgl-1::GFP::3xFLAG]) IV*.
USC1237 – *unc-119(ed3) III; cmpEx93 (myo-3p::GFP, unc-119(+))*.
USC1245 – *mut-16(pk710) I; unc-119(ed3) III; cmpEx93 (myo-3p::GFP, unc-119(+))*

### Strain construction

To generate *cmpEx93(myo-3p::GFP, unc-119(+))*, we injected 5 ng/ul *myo-3p::GFP* (pPD118.20); 10 ng/ul *unc-119(+)* (pCJF151), and 70 ng/ul pBluescript into HT1593 – *unc-119(ed3) III*.

### Brood size assay

Synchronized L1s of wild-type (N2) and *mut-16(pk710)* worms were plated on NGM plates and cultured at 20°C for a single generation. From those plates, L3 hermaphrodites were isolated onto individual plates and cultured at 20°C or 25°C (10 individuals per genotype per temperature). The number of eggs laid, number of eggs hatched and number of progeny reaching L4 stage were counted for each hermaphrodite.

### RNA extraction

Synchronized L1s of wild-type and *mut-16(pk710)* worms were plated on enriched peptone plates and cultured at 20°C and 25°C. Eight thousand animals per sample were harvested as adults (∼68 h at 20°C and ∼48 h at 25°C) for RNA extraction. Worms were washed off plates using water and then settled on ice to form a pellet. Water was aspirated off and worm pellets were resuspended in 1 ml TRIzol reagent (Life Technologies) and freeze-thawed on dry ice followed by vortexing. Worm carcasses were pelleted using centrifugation and the supernatant containing RNA was collected. 0.2 volume chloroform was added to supernatant, vortexed, centrifuged and then the aqueous phase was transferred to a new tube. Samples were precipitated using isopropanol and rehydrated in 50μL nuclease-free H_2_O.

For RNA samples extracted from gonads of wild-type (N2) and *mut-16(pk710)* samples cultured at 20°C and 25°C, 100 adult hermaphrodites were dissected in EBTA buffer with 10% RNAseOUT (ThermoFisher 10777019) then gonads were transferred to TRIzol reagent (Life Technologies) and RNA was extracted as described above. Only the distal arm of each gonad was harvested by cutting at the dorsal-to-ventral bend (10).

### cDNA preparation and qPCR reactions

RNA samples were DNAse treated using TURBO DNAse (ThermoFisher AM2238), and reverse transcribed with SuperScript III Reverse Transcriptase (ThermoFisher 18080093), following manufacturers’ protocols. All Real time PCR reactions were performed using the 2× iTaq Universal SYBER Green Supermix (Biorad 1725121) and run in the CFX96 Touch Real-Time PCR System (Biorad 1855196). Primers used are listed in Supplemental Table S2. qRT-PCR Ct values are reported in Supplemental Table S3.

### mRNA-seq library preparation

Nuclease-free H_2_O was added to 7.5 μg of each RNA sample, extracted from whole animals, to a final volume of 100 μl. Samples were incubated at 65°C for 2 min then incubated on ice. The Dynabeads mRNA Purification Kit (ThermoFisher 61006) was used according to the manufacturer's protocol. 20 μl of Dynabeads were used for each sample. 100 ng of each mRNA sample was used to prepare libraries with the NEBNext Ultra II Directional RNA Library Prep Kit for Illumina (NEB E7760S) according to the manual, using NEBNext multiplex oligos for Illumina (NEB E7335S). Library quality was assessed (Agilent BioAnalyzer Chip) and concentration was determined using the Qubit 1X dsDNA HS Assay kit (ThermoFisher Q33231). Libraries were sequenced on the Illumina NextSeq500 (SE 75-bp reads) platform. Three biological replicates were generated for wild-type (N2) and *mut-16* mutants cultured at 20°C and 25°C.

### Small RNA library preparation

Small RNAs (18- to 30-nt) were size selected on denaturing 15% polyacrylamide gels (Criterion 3450091) from total RNA samples. Libraries were prepared as previously described (53). Library quality was assessed (Agilent BioAnalyzer Chip) and concentration was determined using the Qubit 1X dsDNA HS Assay kit. Libraries were sequenced on the Illumina NextSeq500 (SE 75-bp reads) platform. Three biological replicates were generated for wild-type (N2) and *mut-16* mutants cultured at 20°C and 25°C.

### Isolation of germline nuclei

One million wild-type and *mut-16(pk710)* worms per sample, in biological triplicates, were harvested as adults (∼68 h at 20°C and ∼48 h at 25°C) and germline nuclei were isolated as previously described (54). Worms were washed off plates using M9 media, centrifuged at 3100 rpm for 2 min, and incubated on ice at −20°C for 10 min. Worms were then washed three additional times in M9 media. Worms were crosslinked in 2% formaldehyde in PBS, rotating at room temperature for 30 min, then quenched in a 1 M Tris (pH 7.5) wash. Next, worms were washed two times in M9 media followed by a wash in pre-chilled Nuclei Purification Buffer+ (50 mM HEPES pH 7.5, 40 mM NaCl, 90 mM KCl, 2 mM EDTA, 0.5 mM EGTA, 0.1% Tween-20, 0.5 mM PMSF, 0.2 mM DTT, 0.5 mM spermidine, 0.25 mM spermine, cOmplete protease inhibitor cocktail (Roche)). Worms were resuspended in pre-chilled Nuclei Purification Buffer+ and dounced on ice in a Wheaton homogenizer (clearance 0.05 ± 0.025 nm) for 15 loose strokes followed by 25 tight strokes, with a quarter turn after each stroke. After every 15 strokes, the samples were incubated on ice for 5 min. Twice, the samples were vortexed for 30 s then incubated on ice for 5 min to release the germline nuclei. The nuclei were passed through three 40 μm cell strainers (Fisherbrand) then passed through two 20 μm cell strainers (Pluriselect) to remove worm debris. Isolated nuclei were pelleted at 3100 rpm at 4°C for 6 min and resuspended in Nuclei Purification Buffer+, transferred to a nonstick 1.5 ml tube (Ambion) and an aliquot was DAPI-stained and counted to calculate the number of nuclei extracted. The remainder of the nuclei were pelleted, the supernatant was removed, and the nuclei were flash frozen in liquid nitrogen and stored at −80°C.

### ATAC library preparation

For each sample, roughly 50,000 isolated germline nuclei were used. We prepared our ATAC-seq libraries as previously described (55). Nuclei were resuspended in Nuclei Purification Buffer+ and added to transposition mixture (2× TD Buffer (Nextera), 100 mM Tn5 transposase, PBS, 0.01% digitonin, 0.1% Tween-20) and incubated at 37°C for 30 min, shaking at 1000 rpm. Reactions were cleaned with a Zymo DNA Clean & Concentrator-5 Kit using 1:5 DNA Binding Buffer (Zymo D4014). Transposed fragments were pre-amplified using 2× NEBNext Master Mix (NEB M0541S). Then qPCR amplification was done to determine the number of required additional cycles, using Syber Green I (ThermoFisger Q32851) and 2× NEBNext Master Mix (NEB M0541S), run in the CFX96 Touch Real-Time PCR System (Biorad 1855196). After qPCR, amplification profiles were manually assessed as previously described (56), then samples were amplified accordingly. Libraries were cleaned with a Zymo DNA Clean & Concentrator-5 Kit using 1:5 DNA Binding Buffer (Zymo D4014). Library quality was assessed (Agilent BioAnalyzer Chip) and concentration was determined using the Qubit 1X dsDNA HS Assay kit (ThermoFisher Q32851). The average size of the library was determined with an Agilent Bioanalyzer 2100, and the final pool was quantified via qPCR using the NEBNext Library Quant Kit for Illumina (New England BioLabs, Ipswich, MA, USA), according to manufacturer's instructions. This final pool was sequenced on a high-output flowcell in an Illumina NextSeq 550 instrument, following a 2 × 75 cycles format.

### Bioinformatic analysis

For small RNA libraries and mRNA libraries, sequences were parsed from adapters using Cutadapt (57) and mapped to the *C. elegans* genome, WS258, using HISAT2 (58) and the transcriptome using Salmon (59). Data analysis was done using R, Excel and custom Python scripts. Reads per million were plotted along the WS258 genome using Integrative Genomics Viewer 2.3.68 (60). Gene enrichment analysis was performed using reference gene lists from WormExp (61). CSR-1 target genes, ALG-3/4 target genes, ERGO-1 target genes, *mutator* target genes, piRNA target genes, spermatogenesis-enriched genes, oogenesis-enriched genes, soma-enriched genes, muscle-enriched genes, and neuron-enriched genes were previously described (22,32,37,62–68). For ATAC-seq libraries, adapters were removed using NGMerge (69) and aligned to the WS258 genome using Bowtie2 (70), and analysed using deepTools2 (71). Sequencing data is summarized in Supplemental Table S1.

### Fluorescent microscopy

*C. elegans* gonads were immunostained according to previously described protocol (72). For evaluation of GFP (from *myo-3p::gfp* strain) or PGL-1::GFP::3xFLAG expression and localization, age matched adult animals were imaged using identical microscope settings. To DAPI-stain whole animals, age matched adults were fixed in pre-chilled (−20°C) methanol for 5 min then washed twice in 1xPBST. Worms were then incubated in 0.17 μg/ml DAPI solution (in PBST) for 15 min, followed by three washes in PBST. Imaging was performed on a DeltaVision Elite microscope (GE Healthcare) using a 60× N.A. 1.42 oil-immersion objective. Images were processed using ImageJ (73) and pseudocolored using Adobe Photoshop.

## RESULTS

### Maternal and paternal germline defects contribute to the temperature-sensitive sterility of *mut-16* mutants

**Figure 1.**
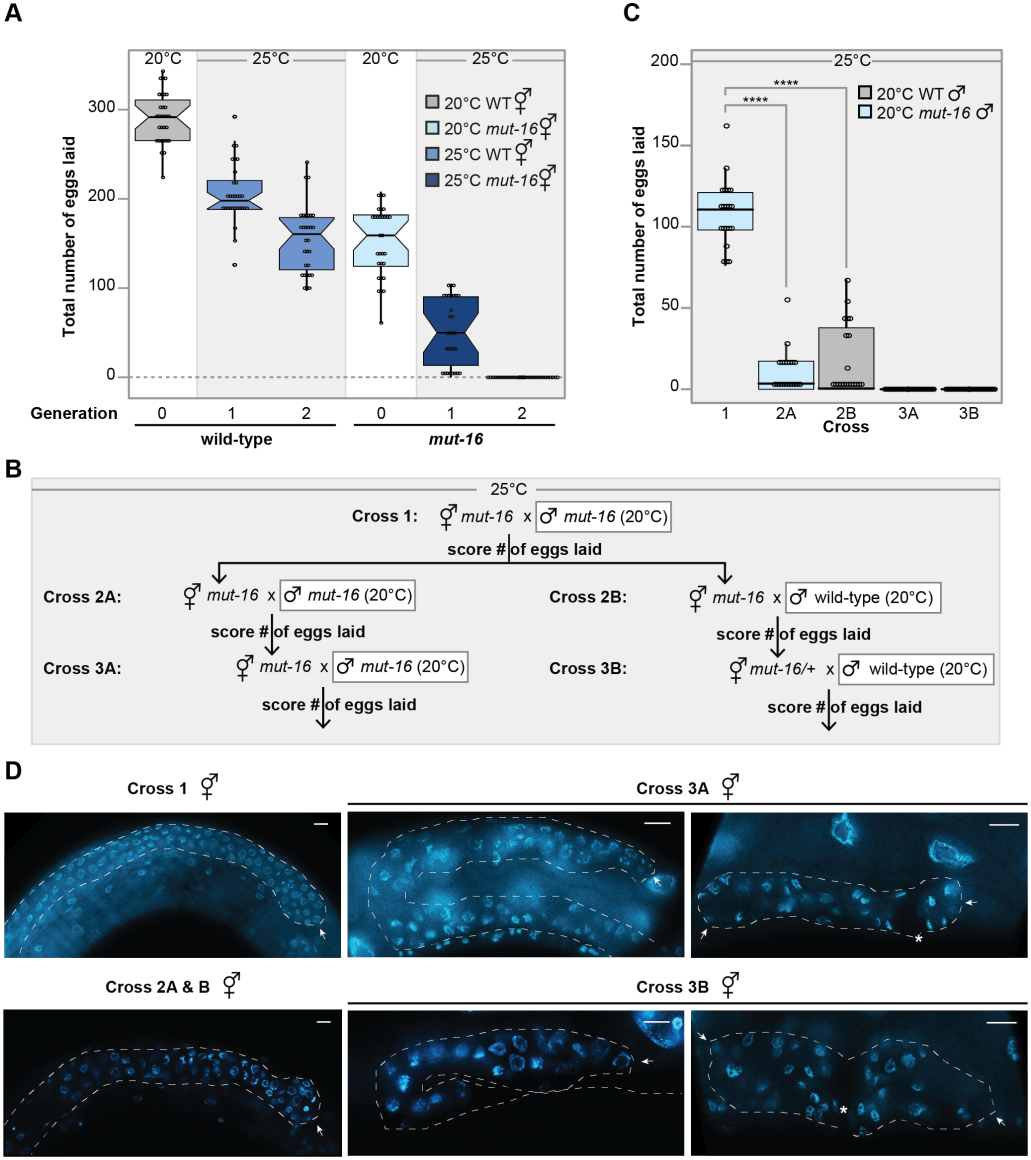
*mut-16* mutants exhibit temperature-sensitive sterility due to maternal and paternal germline defects. (**A**) *mut-16* mutants have reduced brood sizes. Shown are total number of eggs laid by wild-type animals cultured at 20°C (gray), *mut-16* mutants cultured at 20°C (light blue), wild-type animals cultured at 25°C (medium blue), and *mut-16* mutants cultured at 25°C (dark blue). For each generation, *n* = 30 broods. Circles represent each brood counted. Notches indicate the 95% confidence interval of the median; black line indicates median. (**B**) Crossing schema used to assess maternal and paternal effects on *mut-16* fertility at 25°C. All males additionally carried the PGL-1::GFP transgene to confirm successful mating. (**C**) At 25°C, *mut-16* has a maternal effect on fertility that takes two to three generations to manifest. Shown are total number of eggs laid by *mut-16* mutant hermaphrodites grown at 25°C crossed to *mut-16* males grown at 20°C (light blue) or wild-type males grown at 20°C (gray) for each cross shown in the crossing schema. For each generation, *n* = 20 broods. Circles represent each brood counted. Notches indicate the 95% confidence interval of the median; black line indicates median. (**D**) Shown are representative images of DAPI-stained germlines in whole animals of unmated hermaphrodites for each cross shown in the crossing schema. For crosses 3A and 3B, representative images of small germlines are shown in the left panel and representative images of collapsed germlines are shown in the right panel. White dashed line outlines gonad and white arrow indicates distal tip of gonad. Scale bars are 10μm.

Previously, it was shown that *C. elegans* lacking functional MUT-16, like other RNAi-defective mutants, exhibit increased incidence of males and temperature-sensitive sterility when cultured at elevated temperature (37). To further investigate the heat stress-induced sterility in animals lacking functional MUT-16, we performed a multigenerational temperature shift brood size assay using wild-type (N2) and the *mut-16(pk710)* mutant strains. Synchronized animals were cultured at 20°C (Generation 0) and then transferred as L3 larvae to non-permissive temperature (25°C) for subsequent generations (Generations 1–2) (Supplementary Figure S1A). At each generation, we counted the brood size for thirty individuals. Consistent with previous reports, we observed that *mut-16* mutants exhibit a strong temperature-sensitive reduction in fertility (Figure 1A and Supplementary Figure S1B). In addition, we observed that *mut-16* mutants lay on average ∼52% fewer eggs than wild-type animals cultured at permissive temperatures (Figure 1A, Supplementary Figure S1B). We chose to isolate L3s for our brood size analysis due to our observation that *mut-16* mutants exhibited a temperature-independent reduction in progeny viability compared to wild-type animals (Supplementary Figure S1B). It should be noted that when L1s are moved to 25°C, *mut-16* mutants reach sterility within a single generation (37). To take advantage of the complete sterility of *mut-16* mutants in a single generation, we synchronized animals as L1s prior to culturing for all subsequent experiments.

To determine whether the observed temperature sensitive sterility in *mut-16* mutants is due to a maternal or paternal effect of *mut-16*, we performed a multigenerational crossing schema in which we assayed the brood sizes of twenty individual hermaphrodites following mating (Figure 1B). All crosses were performed and maintained at 25°C and males carried the PGL-1::GFP transgene to ensure successful mating. For the first cross, *mut-16* mutant hermaphrodites cultured at 25°C for a single generation, that if unmated would be sterile, were crossed to *mut-16* males cultured at 20°C (cross 1) and the total number of eggs laid were counted (Figure 1B). We found that mating *mut-16* hermaphrodites cultured at 25°C with males that were cultured at permissive temperature restores the fertility of *mut-16* mutant hermaphrodite back to the level similar to *mut-16* hermaphrodites cultured at permissive-temperature (Figure 1C). These data indicate that there is a paternal component to the *mut-16* temperature-sensitive fertility defect. We next mated individual F1 *mut-16* hermaphrodites raised at 25°C from cross 1 to either *mut-16* males cultured at 20°C (cross 2A) or wild-type males cultured at 20°C (cross 2B) (Figure 1B). We observed a significant reduction in total number of eggs laid by F1 *mut-16* hermaphrodites raised at 25°C, compared to the parental generation, independent of whether the F1 *mut-16* hermaphrodites were mated with *mut-16* males (cross 2A) or wild-type males (cross 2B) cultured at 20°C (Figure 1C). These data indicate that there is a maternal fertility defect associated with multiple generations at elevated temperature. Furthermore, when we mated the F2 *mut-16* mutant hermaphrodites to *mut-16* males cultured at 20°C (cross 3A) and the *mut-16/+* heterozygote hermaphrodites to wild-type males cultured at 20°C (cross 3B), neither of the F2 hermaphrodites laid any eggs after mating (Figure 1C). The sterility of cross 3B, the *mut-16/+* heterozygote hermaphrodites crossed to wild-type males, is especially revealing, because it indicates that the sterility of the *mut-16/+* heterozygote hermaphrodite is a maternal effect resulting from its homozygous *mut-16* mutant mother and grandmother being raised at restrictive temperature. To assess the morphology of the *mut-16* mutant female germline after 2–3 generations at elevated temperature, we imaged DAPI-stained germlines of the unmated hermaphrodites used in each of our crosses. Interestingly, the germlines of the *mut-16* mutant hermaphrodites used in cross 3A and cross 3B, neither of which able to produce eggs after mating, exhibited a range of morphological defects (Figure 1D). These defects were observed in all animals and ranged from shrunken and disordered germlines (upper panel) to completely collapsed germlines (lower panel) (Figure 1D). Together, these data demonstrate that *mut-16* mutants have both maternal and paternal fertility defects, however, the paternal fertility defect is manifested within a single generation at elevated temperature, whereas the maternal defect requires 2–3 generations at elevated temperature to induce complete sterility.

### P granule integrity is compromised in *mut-16* mutants at elevated temperature

**Figure 2.**
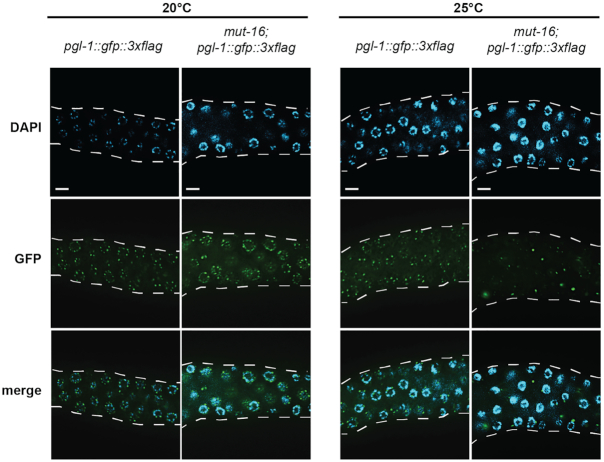
P granules are disrupted in heat-stressed *mut-16* mutants. Expression of PGL-1::GFP::3xFLAG in wild-type and *mut-16* mutant animals cultured at 20°C and 25°C. White dashed line outlines gonad. Scale bars are 10 μm.

Because expression of PGL-1, a core component of P granules, is restricted to the germline, it is often used to evaluate the identity of germline versus somatic tissue (74,75). To determine whether the loss of fertility we observe in heat-stressed *mut-16* mutants is accompanied by loss of P granules, we evaluated the expression of PGL-1::GFP::3xFLAG in the germline of wild-type and *mut-16* mutant animals cultured at 20°C and 25°C. As expected, wild-type animals cultured at 20°C and 25°C express PGL-1::GFP::3xFLAG in normal perinuclear P granules (Figure 2). In addition, we observed normal perinuclear P granules containing PGL-1::GFP::3xFLAG in *mut-16* mutants cultured at 20°C (Figure 2). Strikingly, PGL-1::GFP::3xFLAG expression in *mut-16* mutants cultured at 25°C was severely disrupted, with some germline nuclei flanked by large aggregates of PGL-1::GFP::3xFLAG whereas other nuclei in the same germline completely lacked perinuclear P granules (Figure 2). These data indicate P granule integrity is disrupted in heat-stressed *mut-16* mutants.

### Spermatogenesis-enriched genes are up-regulated during heat stress

**Figure 3.**
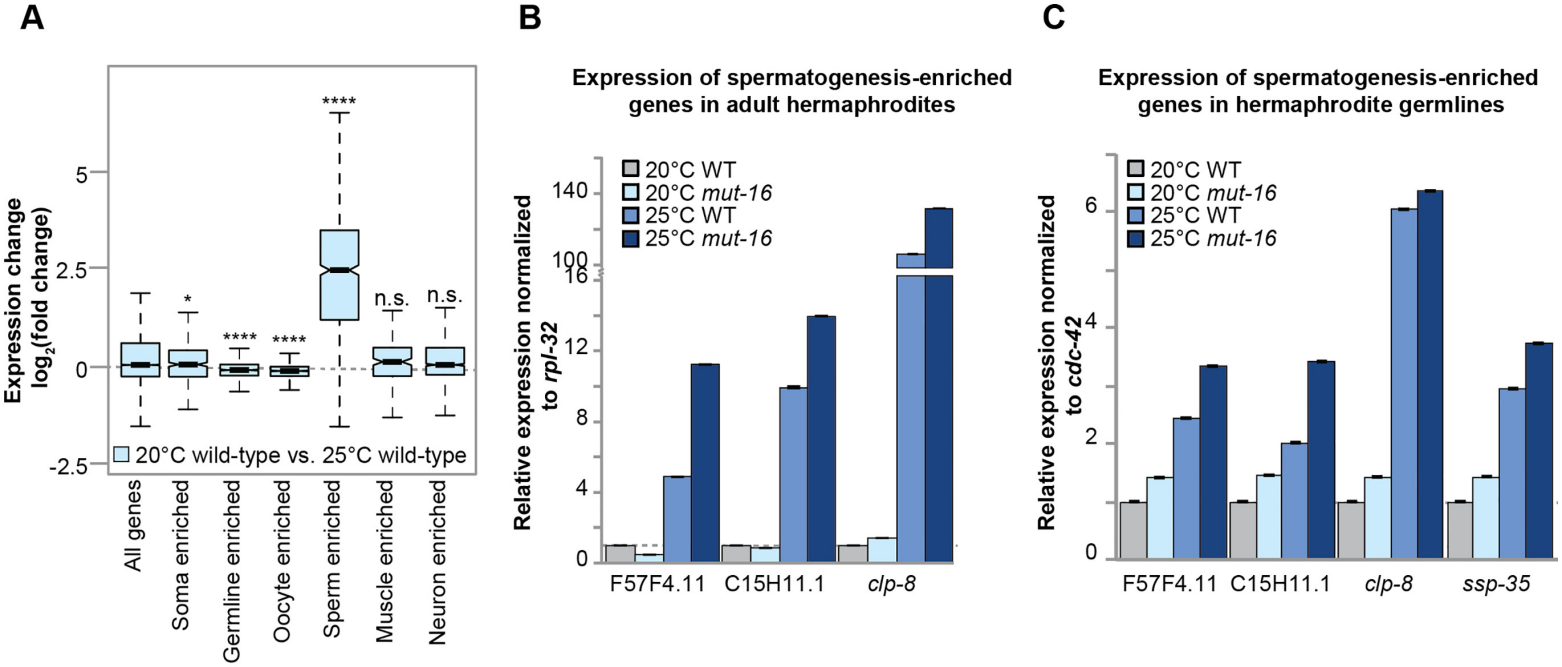
Heat stress induces expression of spermatogenesis-enriched genes in adult hermaphrodites. (**A**) Comparison of expression changes in wild-type animals cultured at 25°C compared to wild-type animals cultured at 20°C for published enriched gene sets. Notches indicate the 95% confidence interval of the median; black line indicates median. n.s. denotes not significant and indicates a *P*-value > 0.05, * indicates a *P*-value ≤ 0.05, **** indicates a *P*-value ≤ 0.0001. (**B**) qRT-PCR of spermatogenesis factors in hermaphrodites of wild-type and *mut-16* mutant animals cultured at 20°C and 25°C. Expression is normalized to *rpl-32* and relative to expression in wild-type animals cultured at 20°C. Error bars indicate standard deviation. (**C**) qRT-PCR of spermatogenesis factors in the germlines of hermaphrodites of wild-type and *mut-16* mutant animals cultured at 20°C and 25°C. Expression is normalized to *cdc-42* and relative to expression in wild-type animals cultured at 20°C. Error bars indicate standard deviation.

In small RNA pathway mutants that exhibit a temperature-sensitive mortal germline phenotype, the generation at which sterility occurs differs (12,37,41–43). As it has remained unclear what triggers induction of sterility, we sought to identify mRNA expression changes that occur during heat stress that may contribute to the reproductive changes in temperature-sensitive small RNA pathway mutants. We generated mRNA-seq libraries from synchronized adult wild-type and *mut-16* mutants cultured at 20°C and 25°C for a single generation (Supplementary Table S1). In addition, we sequenced size selected small RNA libraries from the total RNA extracted from synchronized adult wild-type and *mut-16* mutants cultured at 20°C and 25°C for a single generation (Supplementary Table S1). We used differential expression analysis to identify genes that exhibit transcript level changes upon heat stress. First, we compared mRNA libraries from wild-type animals cultured at 20°C to mRNA libraries generated from wild-type animals cultured at 25°C to identify genes significantly differentially expressed upon heat stress. This analysis revealed that spermatogenesis-enriched genes were significantly up-regulated in wild-type animals during heat stress (Figure 3A). Similarly, when we compared mRNA libraries from *mut-16* mutant animals cultured at 20°C to mRNA libraries generated from *mut-16* mutant animals cultured at 25°C, we observed a significant up-regulation of spermatogenesis-enriched genes (Supplementary Figure S2A). In total, we found 1478 genes were significantly up-regulated and 121 genes were significantly down-regulated upon heat stress in both wild-type and *mut-16* mutant animals. Further bioinformatic analysis revealed that spermatogenesis factors are significantly enriched among the 1478 genes significantly up-regulated (35% of up-regulated genes, enrichment analysis log_2_(fold change) = 2.56), while oogenesis factors and gender-neutral gonad factors were significantly depleted among the up-regulated genes (enrichment analysis log_2_(fold change) = −5.80 and log_2_(fold change) = −0.71, respectively) (Supplementary Figure S2B). In addition, 28% of the 121 genes that are down-regulated during heat stress are associated with oogenesis or gender-neutral germline function (Supplementary Figure S2C). These data indicate that heat-stressed animals display increased expression of spermatogenesis-associated transcripts and reduced expression of oogenesis and other germline transcripts.

Incidence of males in wild-type *C. elegans* cultured at permissive temperature is incredibly low (∼0.1%), however the incidence of males increases when cultured at non-permissive temperatures (∼0.4%) (37,76–79). In addition, *mut-16* mutants have been observed to have an increased incidence of males independent of temperature (2.1% at permissive temperature and 5.9% at non-permissive temperature) (37). To determine whether the observed enrichment of spermatogenesis factors among heat-induced genes could be attributed to an increased incidence of males in the prepared mRNA libraries, we performed qRT-PCR using RNA extracted from isolated hermaphrodites of wild-type and *mut-16* mutants grown at 20°C and 25°C. We used primers for highly up-regulated spermatogenesis factors from our list of heat-induced genes (F57F4.1, C15H11.1 and *clp-8*). We found that these spermatogenesis factors up-regulated due to heat stress in our mRNA-seq libraries were also up-regulated by qRT-PCR of adult hermaphrodites, indicating that the increase in expression of spermatogenesis genes cannot be attributed to an increased incidence of males in the mRNA-seq samples (Figure 3B). While culturing animals at elevated temperatures is known to cause higher incidence of male progeny, our data indicates that heat stress also results in increased expression of male-specific and spermatogenesis-enriched factors in adult hermaphrodites. Our mRNA-seq libraries were generated from RNA extracted from whole animals. Next, to determine whether the observed increase in expression of spermatogenesis factors in heat-stressed animals is occurring in germline tissue, we performed qRT-PCR using RNA extracted from dissected hermaphrodite germlines of wild-type and *mut-16* mutants grown at 20°C and 25°C. Samples contained only the distal arms of the gonads so as to exclude oocytes and the sperm. We observed a significant increase in expression of the tested spermatogenesis factors in the germlines of heat-stressed animals (Figure 3C). Together, these data reveal that spermatogenesis factors are mis-regulated within the germlines of heat-stressed hermaphrodites.

### Spermatogenic and somatic genes are up-regulated in heat-stressed *mut-16* mutants

**Figure 4.**
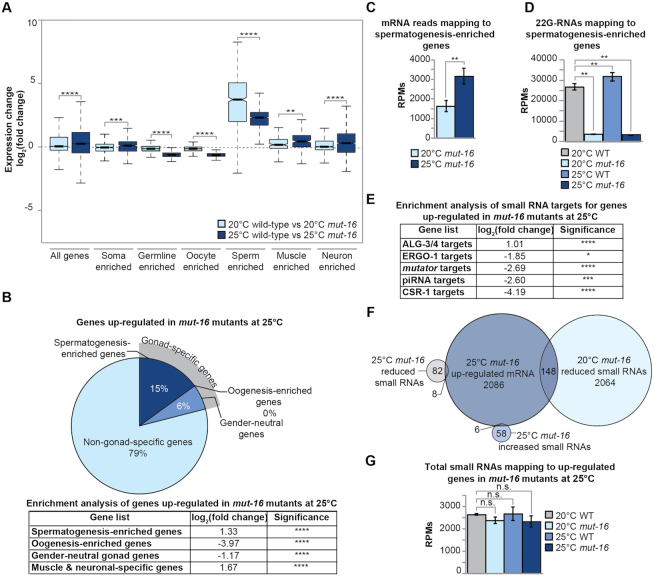
Expression changes for protein-coding genes in heat-stressed *mut-16* mutants. (**A**) Comparison of expression changes in wild-type animals cultured at 20°C compared to *mut-16* mutants cultured at 20°C (light blue) and wild-type animals cultured at 25°C compared to *mut-16* mutants cultured at 25°C (dark blue) for published enriched gene sets. Notches indicate the 95% confidence interval of the median; black line indicates median. Significance between the log_2_(fold change) at 20°C compared to the log_2_(fold change) at 25°C is indicated. (**B**) Percentages of gonad-specific and non-gonad-specific genes represented in the genes up-regulated exclusively in *mut-16* mutants at 25°C, compared to wild-type animals at 20°C and 25°C and *mut-16* mutants at 20°C. Enrichment analysis for spermatogenesis, oogenesis, gender-neutral genes, and muscle-specific and neuronal-specific genes amongst the genes up-regulated during heat stress in *mut-16* mutants is in the table below. (**C**) mRNA transcripts mapping to spermatogenesis-enriched genes are counted, in reads per million (RPMs), for *mut-16* mutants cultured at 20°C and 25°C. Error bars indicate standard deviation. (**D**) 22G-RNAs mapping to spermatogenesis-enriched genes are counted, in reads per million (RPMs), for wild-type and *mut-16* mutants cultured at 20°C and 25°C. Error bars indicate standard deviation. (**E**) Enrichment analysis for small RNA pathway target genes represented in the genes up-regulated exclusively in *mut-16* mutants at 25°C. (**F**) Venn diagram of genes up-regulated exclusively in *mut-16* mutants at 25°C at the mRNA level (dark blue), genes with increased levels of total small RNAs exclusively in *mut-16* mutants at 25°C (medium blue), genes with decreased levels of small RNAs in *mut-16* mutants at 20°C (light blue), and genes with decreased levels of small RNAs exclusively in *mut-16* mutants at 25°C (grey). (**G**) Total small RNAs mapping to the genes up-regulated exclusively in *mut-16* mutants at 25°C are counted, in reads per million (RPMs), for wild-type and *mut-16* mutants cultured at 20°C and 25°C. Error bars indicate standard deviation. n.s. denotes not significant and indicates a *P*-value > 0.05, * indicates a *P*-value ≤ 0.05, ** indicates a *P*-value ≤ 0.01, *** indicates a *P*-value ≤ 0.001, **** indicates a *P*-value ≤ 0.0001.

To further understand the changes in expression associated with the fertility defects of *mut-16* mutants at elevated temperature, we used differential expression analysis to identify the genes that are significantly down-regulated or up-regulated (519 genes and 2086 genes, respectively) exclusively in *mut-16* mutants cultured at 25°C. These gene lists exclude any genes we determined to be significantly differentially expressed due to heat stress or the *mut-16* mutation alone. This analysis revealed that soma-enriched genes, muscle-enriched genes, neuron-enriched genes and sperm-enriched genes were significantly up-regulated in *mut-16* mutants cultured at 25°C, compared to both *mut-16* mutants cultured at 20°C and wild-type animals cultured at 25°C (Figure 4A and B). It should be noted that the log_2_(fold change) of spermatogenesis-enriched genes in *mut-16* mutants cultured at 25°C does not appear significantly up-regulated compared to *mut-16* mutants cultured at 20°C due to the significant up-regulation of spermatogenesis-enriched genes present in wild-type animals cultured at 25°C compared to wild-type animals cultured at 20°C (Figure 3A and Supplementary Figure S2A). In fact, there is significant increased expression of spermatogenesis-enriched genes in *mut-16* mutants cultured at 25°C, compared to *mut-16* mutants cultured at 20°C (Figure 4C). Interestingly, our analysis revealed that 22G-RNAs mapping to spermatogenesis-enriched genes are depleted in *mut-16* mutants, correlating with increased expression of the genes, but 22G-RNAs are not significantly changed in wild-type (N2) animals cultured at 25°C, compared to wild-type (N2) animals cultured at 20°C (Figure 4D). This suggests that the expression of spermatogenesis-enriched genes is susceptible to changes in small RNAs and heat stress in an independent manner.

We next focused on the genes that are significantly up-regulated exclusively in *mut-16* mutants at elevated temperature, which were enriched for non-gonad-specific genes (79%) such as muscle-specific and neuronal-specific genes (enrichment analysis log_2_(fold change) = 1.67) (Figure 4A and B). Because MUT-16 is critical for the biogenesis of WAGO-class 22G-siRNA pathway, we sought to determine if any of the up-regulated genes are direct targets of a known small RNA pathway. Interestingly, the majority of the genes significantly up-regulated exclusively in the heat-induced sterile generation of *mut-16* mutants are not known targets of any small RNA pathway (94%). However, there was enrichment for targets of the ALG-3/4 pathway amongst the up-regulated genes (log_2_(fold change) = 1.01), whereas there was significant under-representation of ERGO-1 targets (log_2_(fold change) = −1.85), *mutator* targets (log_2_(fold change) = −2.69), piRNA targets (log_2_(fold change) = −2.60), and CSR-1 targets (log_2_(fold change) = −4.19) (Figure 4E).

To determine whether the genes up-regulated in *mut-16* mutants experiencing heat stress could be explained by changes in small RNA populations, we compared the small RNA libraries generated from synchronized adult wild-type and *mut-16* mutants cultured at 20°C and 25°C for a single generation (Supplementary Table S1). After bioinformatic analysis, we found that 140 genes were differentially targeted by small RNAs exclusively in the *mut-16* mutants cultured at 25°C. This list excluded genes that were differentially targeted due to heat stress or to the *mut-16* mutation alone. Of the 140 genes differentially targeted by small RNAs, 82 genes had significant reduction of small RNAs in *mut-16* mutants grown at 25°C compared to *mut-16* mutants grown at 20°C and wild-type animals (20°C and 25°C) and 58 genes had a significant increase of small RNAs mapping to the gene in *mut-16* mutants grown at 25°C compared to *mut-16* mutants grown at 20°C and wild-type animals (20°C and 25°C). Of the 2,086 genes significantly up-regulated exclusively in *mut-16* mutants cultured at elevated temperature, only 14 overlapped with small RNA targets with significant differential expression exclusively in the *mut-16* mutants cultured at 25°C (Figure 4F). A portion of the genes significantly up-regulated at the transcript level in *mut-16* mutants cultured at 25°C (148 of the 2086 genes) corresponded to genes with reduced small RNA reads in *mut-16* mutants at permissive temperature (20°C) (Figure 4F). Overall, there was not a significant change in total small RNA levels mapping to the up-regulated genes in heat-stressed *mut-16* mutants (Figure 4G). This data indicates that the change in transcript level of these genes is not directly regulated by changes in small RNAs at elevated temperatures, but some genes that lose small RNAs at permissive temperature (20°C) in RNAi pathway mutants have increased expression once the animals experience heat stress. Therefore, our analysis indicates that, in *mut-16* mutants, heat stress triggers an increase in the expression of soma-specific genes in a small RNA-independent manner.

### Oogenesis and germline genes are down-regulated in heat-stressed *mut-16* mutants

When we examined the genes that are significantly down-regulated in *mut-16* mutants cultured at 25°C compared to *mut-16* mutants cultured at 20°C, we found primarily germline-enriched genes (Figure 4A and Supplementary Figure S3A). Specifically, down-regulated genes in the heat stressed *mut-16* mutants were enriched for oogenesis-enriched genes and gender-neutral gonad-specific genes (log_2_(fold change) = 1.90 and log_2_(fold change) = 1.60, respectively) (Supplementary Figure S3A). Furthermore, enrichment analyses revealed that these down-regulated genes are significantly enriched for CSR-1 targets (log_2_(fold change) = 2.13) and mildly enriched for ALG-3/4 targets (log_2_(fold change) = 0.65) in wild-type animals (Supplementary Figure S3B). This data indicates that the heat-induced sterility of *mut-16* mutants is associated with reduced expression of oogenic and gender-neutral gonad-specific genes on the transcript level and that this repression may be due to mis-regulation of CSR-1-associated small RNAs that typically protect the transcriptional status of these genes’ expression or a more general loss of germ cell identity.

### Increased transposon activity is not the underlying cause of sterility in heat-stressed *mut-16* mutants

**Figure 5.**
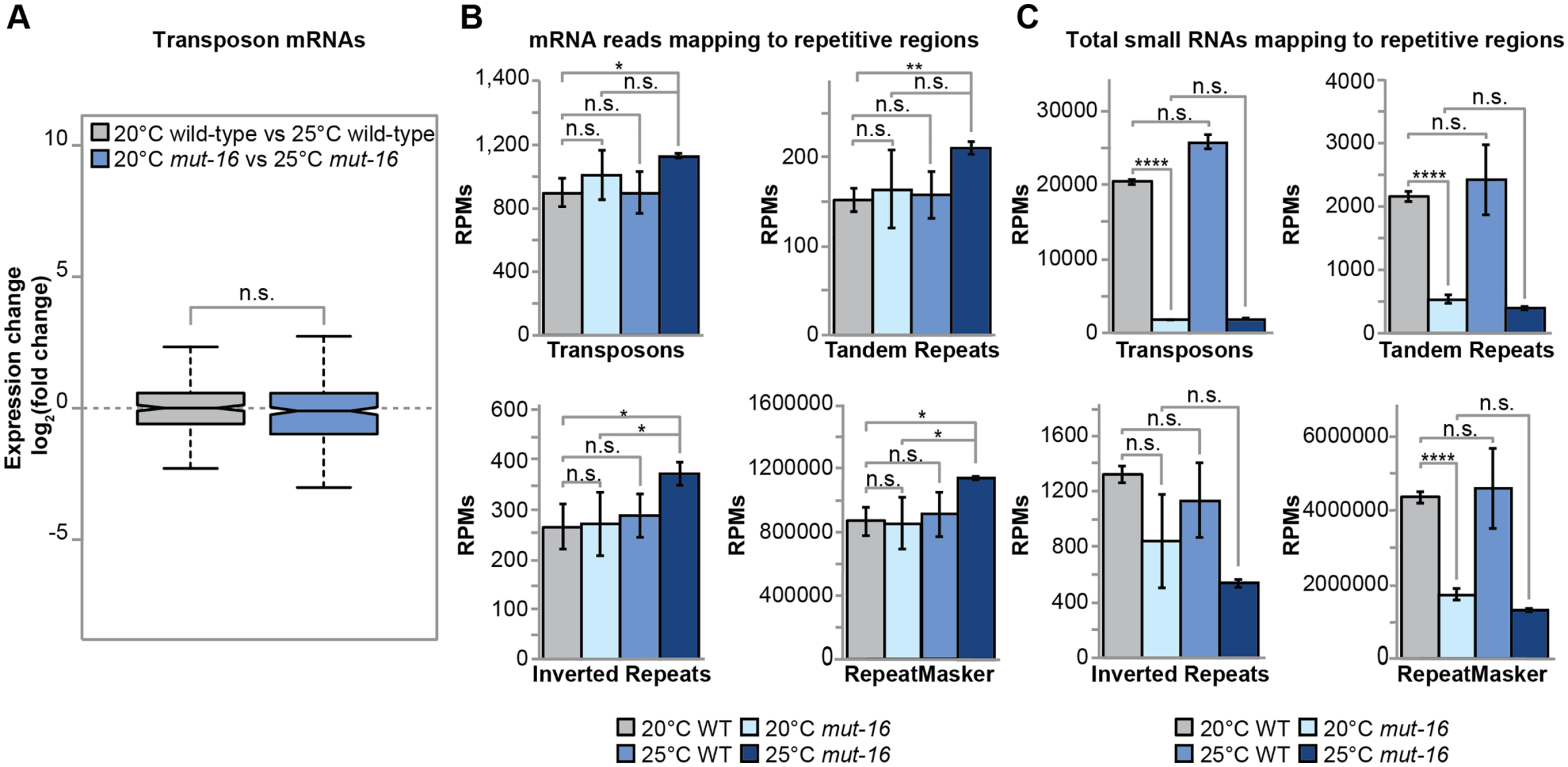
Repetitive element mRNA expression is only modestly affected by heat-stress in *mut-16* mutants. (**A**) Comparison of transposon mRNAs expression changes in wild-type animals cultured at 20°C compared to 25°C (gray) and *mut-16* mutants cultured at 20°C compared to 25°C (blue). Notches indicate the 95% confidence interval of the median; black line indicates median. (**B**) Shown are reads per million (RPMs) from mRNA-seq libraries mapping to annotated transposons, tandem repeats, inverted repeats, and the RepeatMasker of WS258 for wild-type and *mut-16* mutants cultured at 20°C and 25°C. Error bars indicate standard deviation. (**C**) Shown are reads per million (RPMs) from small RNA libraries mapping to annotated transposons, tandem repeats, inverted repeats, and the RepeatMasker of WS258 for wild-type and *mut-16* mutants cultured at 20°C and 25°C. Error bars indicate standard deviation. n.s. denotes not significant and indicates a *P*-value > 0.05, * indicates a *P*-value ≤ 0.05, ** indicates a *P*-value ≤ 0.01, **** indicates a *P*-value ≤ 0.0001.

One proposed cause of sterility in heat-stressed RNAi-defective mutants is increased transposition of transposable elements (TEs) that would result in accumulation of DNA damage and increased expression of TEs. We assessed differential expression of annotated transposable elements (TEs), tandem repeats, and inverted repeats in the heat-induced sterile generation of *mut-16* mutants. We found the expression of TE mRNAs are not significantly changed in *mut-16* mutants cultured at 25°C, compared to *mut-16* mutants cultured at 20°C (Figure 5A and B). In addition, we observed only a modest increase in the expression of tandem repeats mRNAs, inverted repeats mRNAs, and general RepeatMasker annotated transcripts, which include simple repeats and low complexity regions, in *mut-16* mutants cultured at 25°C, compared to *mut-16* mutants cultured at 20°C (Figure 5B). Furthermore, small RNAs mapping to TEs, tandem repeats, and general RepeatMasker annotated regions were depleted in *mut-16* mutants cultured at 20°C, but were not further depleted in *mut-16* mutants upon heat stress (Figure 5C). Our results are consistent with a recent study that looked at the expression of TE mRNAs in heat-stressed *prg-1(n4357)* and *mut-16(pk710)* mutants (80). These observations support the conclusion that accumulation of DNA damage due to an increase in TE transposition is not the underlying cause of sterility in heat-stressed RNAi-defective animals.

### Up-regulation of somatic genes is a common feature of heat-stressed small RNA pathway mutants

**Figure 6.**
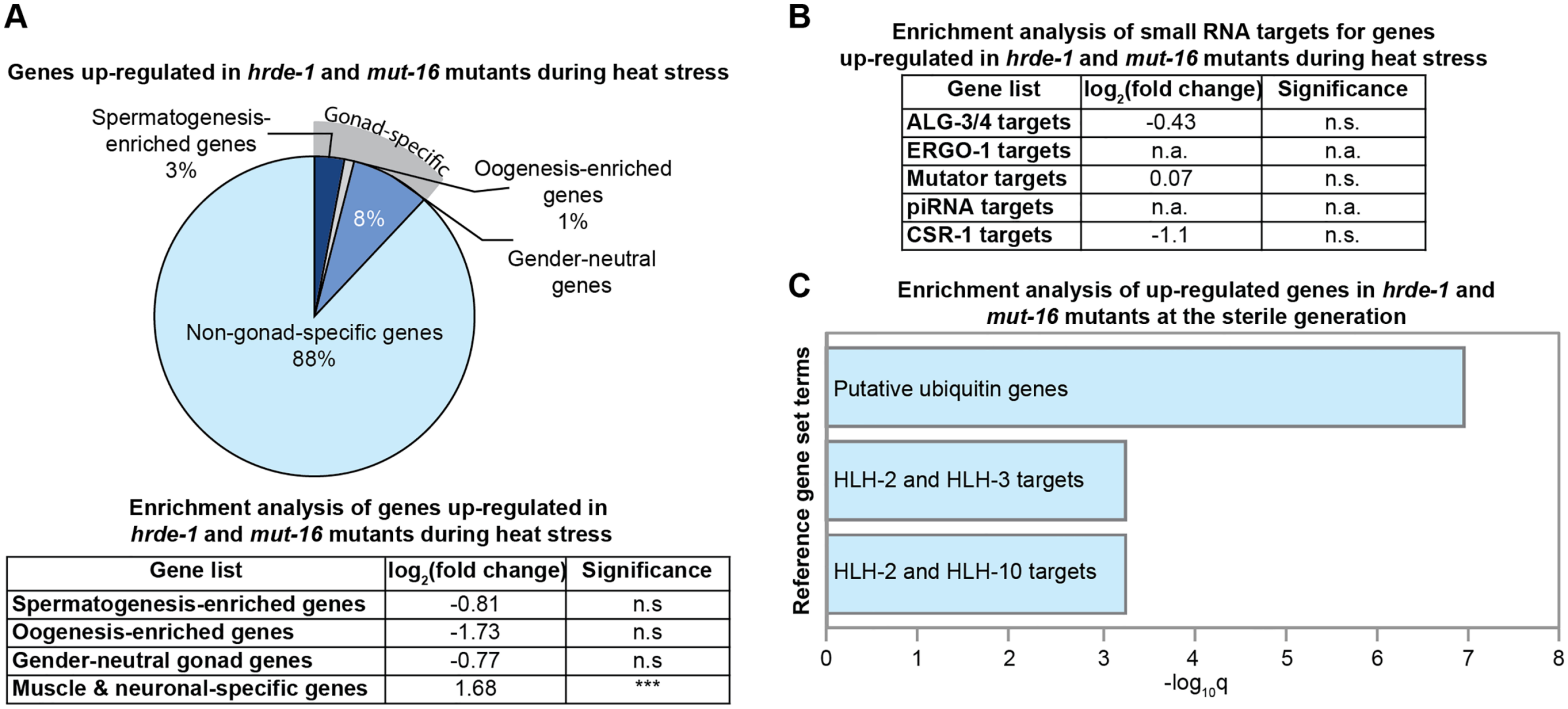
Somatic genes are aberrantly expressed in heat-stressed *mut-16* and *hrde-1* mutants. (**A**) Percentages of gonad-specific and soma-specific genes represented in the genes specifically up-regulated in *mut-16* and *hrde-1* mutants at 25°C. Enrichment analysis for spermatogenesis, oogenesis, gender-neutral genes, and muscle-specific and neuronal-specific genes among the genes up-regulated in both *mut-16* and *hrde-1* mutants at elevated temperature is shown in the table below. n.s. denotes not significant and indicates a *P*-value > 0.05 and *** indicates a *P*-value ≤ 0.001. (**B**) Enrichment analysis for small RNA pathway target genes represented in the genes up-regulated in both *mut-16* and *hrde-1* mutants at elevated temperature. n.a. denotes ‘not applicable’ when zero genes were represented. n.s. denotes not significant and indicates a *P*-value > 0.05. (**C**) Gene enrichment analysis was performed using WormExp (log_10_Q ≥ 2 and FDR < 0.05) for genes up-regulated in *mut-16* and *hrde-1* mutants at 25°C. See Supplementary Figure S4 for complete list of enriched gene sets.

We next sought to address whether the changes in expression observed in heat-stressed *mut-16* mutants are a common feature of small RNA pathway mutants raised at elevated temperature. We utilized previously published mRNA-seq libraries from adult wild-type and *hrde-1(tm1200)* mutant strains cultured at 15°C for three generations and 23°C for six additional generations (42). We bioinformatically compared the lists of genes significantly down-regulated and up-regulated in both the *mut-16* mutant and *hrde-1* mutant libraries after culturing at elevated temperatures (a single generation at 25°C for *mut-16* and six generations at 23°C for *hrde-1*). We identified 8 genes significantly down-regulated and 88 genes significantly up-regulated during heat stress in both *mut-16* and *hrde-1* mutants. Interestingly, we found that the genes significantly up-regulated in the sterile generation of both *mut-16* and *hrde-1* mutants were predominantly somatic genes (88%) and that these genes were enriched for muscle-specific and neuron-specific genes (log_2_(fold change) = 1.68) (Figure 6A). Surprisingly, the genes significantly up-regulated in the sterile generation of both *mut-16* and *hrde-1* mutants were not enriched for known RNAi pathway targets (Figure 6B). We performed enrichment analysis using reference gene lists (TF targets, Tissue, and Other) from WormExp (61) (log_10_Q ≥ 2 and FDR < 0.05) and found the list of genes up-regulated in the heat-induced sterile generations of both *hrde-1* and *mut-16* mutants was enriched for genes encoding putative ubiquitin ligase components (Figure 6C and Supplementary Figure S4). Furthermore, these genes were enriched for F-box factors or proteins predicted to contain an F-box domain (87% of the up-regulated genes). F-box factors provide the specificity of the Skip-Cullin-F-box (SCF) E3 ubiquitin ligase complex for target proteins to be ubiquitinated and subsequently degraded by the proteasome. In addition, the genes up-regulated in RNAi pathway mutants during heat stress were enriched for somatically-expressed targets of evolutionarily conserved Helix-Loop-Helix (HLH) transcription factors, despite no observed change in the expression levels of most HLH transcription factors (Figure 6C and Supplementary Figure S4). These data indicate that heat stress triggers increased expression of somatic genes and F-box factor components of the SCF E3 ubiquitin ligase complex in RNAi defective mutants.

### Heat-stressed *mut-16* mutants mis-express somatic transcripts in germ cells

**Figure 7.**
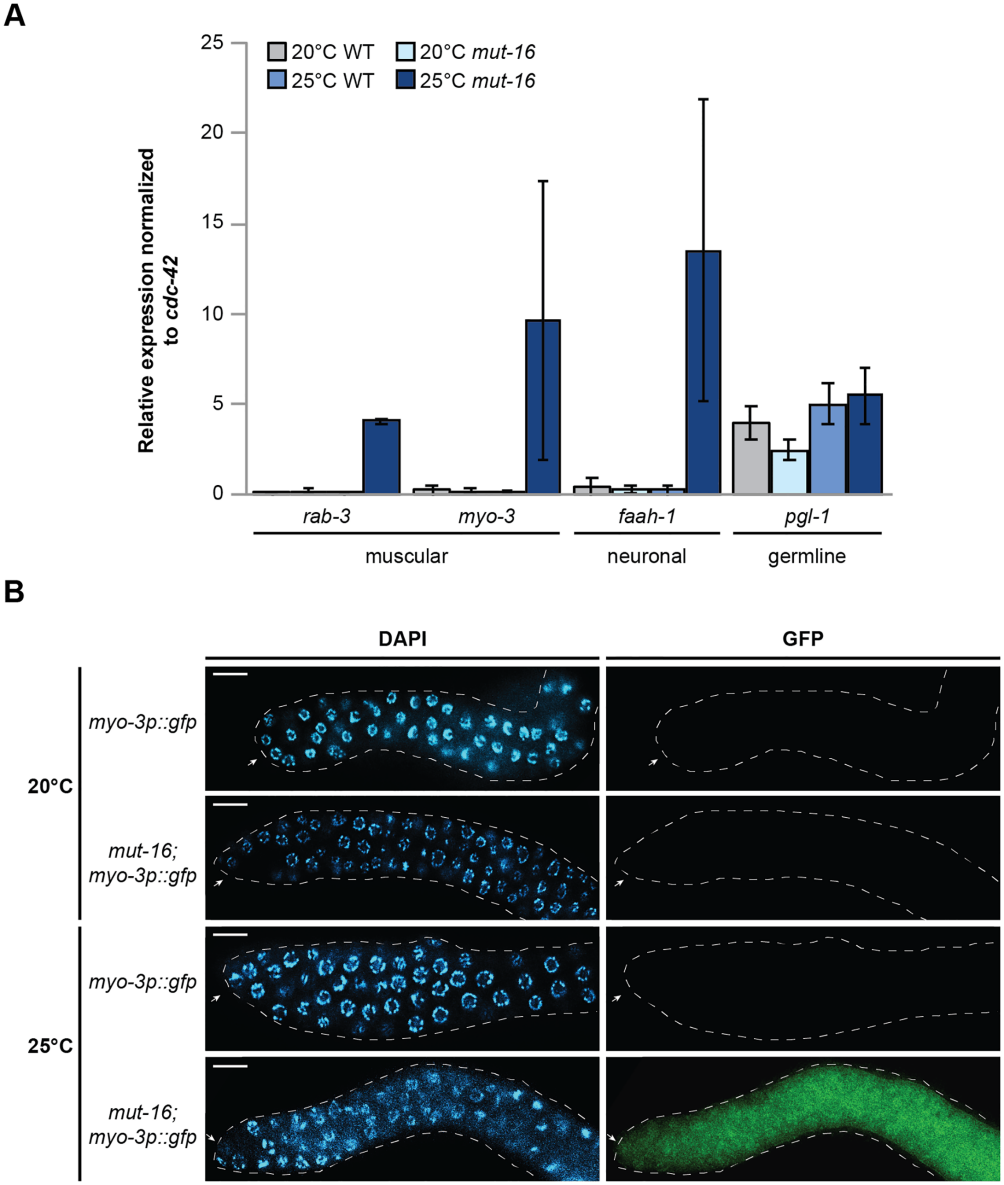
Germ cells aberrantly express somatic cell markers in *mut-16* mutants cultured at 25°C. (**A**) qRT-PCR of genes expressed in muscle and neuronal cells in gonads of wild-type and *mut-16* mutants cultured at 20°C and 25°C. Expression is normalized to *cdc-42*. *pgl-1* is used as a control for sample specificity. Error bars indicated standard deviation. (**B**) Expression of the *myo-3p::GFP* transgene in dissected gonads of wild-type and *mut-16* mutants cultured at 20°C and 25°C. White dashed line outlines gonad and white arrow indicates distal tip of gonad. Scale bars are 10 μm.

Previously, it was shown that inappropriate expression of somatic genes occurs in the germline as a result of P granule disruption, or loss of translational regulators or chromatin-modifying enzymes (1–4,11). Because the observed increase in expression of soma-specific genes in the heat-stressed *mut-16* and *hrde-1* mutants could be occurring in either somatic tissues or germline tissues, we sought to determine whether we could observe an increase in soma-specific gene expression in isolated germline tissue. To this end, we used RNA from isolated gonad tissue from synchronized adult wild-type and *mut-16* mutants cultured at 20°C and 25°C for a single generation, to perform qRT-PCR for muscle (*rab-3* and *myo-3*) and neuronal (*faah-1*) markers that were significantly up-regulated in the mRNA libraries from heat stressed *mut-16* mutants. We used *pgl-1* as a control for the specificity of our gonad-extracted RNA. It should be noted, that despite the disruption of P granule integrity in *mut-16* mutants grown at 25°C (Figure 2), we did not observe a change in *pgl-1* expression, or the expression of other core P granule components, in our mRNA-seq libraries for wild-type and *mut-16* mutants grown at 20°C and 25°C (Supplementary Figure S5A and B). We found that the muscular and neuronal markers were significantly up-regulated in the isolated germline tissue of heat-stressed *mut-16* mutants (25°C), indicating that germline cells are indeed ectopically expressing somatic transcripts (Figure 7A).

As a complementary approach to the qRT-PCR and to further validate the ectopic expression of somatic genes in the germline of heat-stressed *mut-16* mutants, we cultured wild-type and *mut-16* animals carrying a transgene array expressing GFP driven by the promoter of *myo-3* at 20°C and 25°C. *myo-3*, which was up-regulated in our mRNA-seq data as well as in the germline RNA qRT-PCR, is a marker of body wall muscle and is not expressed in the *C. elegans* gonad under normal conditions. As expected, wild-type animals cultured at 20°C and 25°C did not express *myo-3p::GFP* within their gonad (Figure 7B). *myo-3p::GFP* was also not expressed in the gonad of *mut-16* mutants cultured at 20°C; however, *myo-3p::GFP* was strongly expressed in the gonad of *mut-16* mutants cultured at 25°C (Figure 7B). The results of the qRT-PCR and observation of *myo-3* promoter-driven GFP expression in the gonads at 25°C indicates that *mut-16* mutants at elevated temperature are inappropriately expressing soma-specific genes within the gonad tissues. These data suggest that the germ cells of heat-stressed *mut-16* mutants lose the ability to repress somatic transcripts such as *myo-3*.

### Up-regulation of somatic transcripts in the germline of heat-stressed *mut-16* mutants corresponds with global changes in germline chromatin accessibility

**Figure 8.**
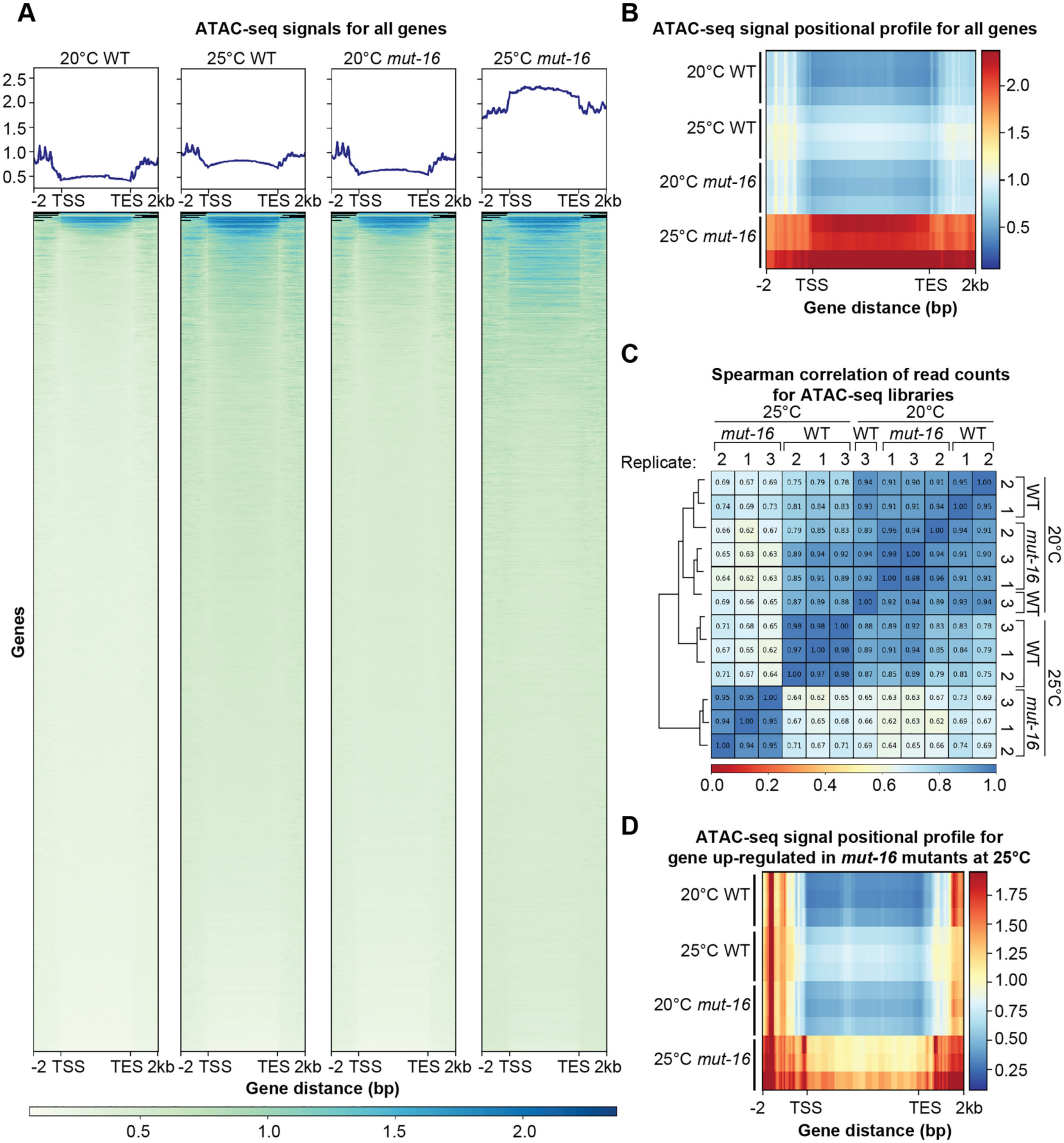
Heat stress in *mut-16* mutants leads to increased chromatin accessibility. (**A**) Normalized plots and heat maps of ATAC-seq signals for each annotated gene in the WS258 genome. Each gene body is scaled to 5 kb and includes 2 kb upstream of the transcription start site (TSS) and 2 kb downstream of the transcription end site (TES). Blue represents high chromatin accessibility and white represents low chromatin accessibility. One representative biological replicate is shown for each genotype and temperature. (**B**) Average positional profile heat map of ATAC-seq signals for each annotated gene in the WS258 genome. Each gene body is scaled to 5 kb and includes 2 kb upstream of the transcription start site (TSS) and 2 kb downstream of the transcription end site (TES). Red represents high chromatin accessibility and dark blue represents low chromatin accessibility. Data for all three biological replicates are shown. (**C**) Heat map of the Spearman correlation coefficient for each ATAC-seq library. Blue is high correlation and red is no correlation. (**D**) Average positional profile heat map of ATAC-seq signals for genes up-regulated exclusively in *mut-16* mutants at 25°C. Each gene body is scaled to 5 kb and includes 2 kb upstream of the transcription start site (TSS) and 2 kb downstream of the transcription end site (TES). Red represents high chromatin accessibility and dark blue represents low chromatin accessibility. Data for all three biological replicates are shown.

RNAi can regulate mRNAs either post-transcriptionally or transcriptionally by directing the establishment of the repressive chromatin mark H3K9me3 at target loci (12,13,16,17,29). Because the majority of genes up-regulated significantly in the heat stressed *mut-16* mutants are not known targets of a RNAi pathway, we sought to determine whether changes in the chromatin structure correlates with increased expression of the somatic genes in the germlines of heat-stressed *mut-16* mutants. To this end, we isolated germline nuclei from synchronized adult wild-type and *mut-16* mutants cultured at 20°C and 25°C for a single generation and generated Assay for Transposase-Accessible Chromatin (ATAC)-seq libraries. Our bioinformatic analysis revealed that there is increased chromatin accessibility globally in the germline nuclei of heat-stressed wild-type animals compared to wild-type cultured at permissive temperatures (Figure 8A and B). The increased global chromatin accessibility is further exacerbated in heat-stressed *mut-16* mutants compared to wild-type animals, grown at 20°C and 25°C, and *mut-16* mutants grown at 20°C (Figure 8A and B). We calculated the Spearman correlation coefficient and performed principle component analysis to assess the similarities of our ATAC-seq libraries, and found that while the samples for wild-type and *mut-16* mutants grown at 20°C are very similar, the samples for *mut-16* mutants grown at 25°C are completely dissimilar from the other samples (Figure 8C and Supplementary Figure S6). These data indicate that heat-stress results in global changes in chromatin accessibility along gene bodies in *C. elegans*, and that *mut-16* mutants are more susceptible to changes in chromatin accessibility upon heat stress.

Next, we assessed the chromatin accessibility of genes that are up-regulated exclusively upon heat-stress in *mut-16* mutants. We found a mild increase in chromatin accessibility along the bodies of these genes in heat-stressed wild-type animals and a drastic increase in chromatin accessibility along the bodies of these genes in heat-stressed *mut-16* mutants (Figure 8D). Interestingly, we also found that the bodies (transcription start site (TSS) to transcription end site (TES)) of these genes are flanked by regions of accessible chromatin (Figure 8D). Together, these data show that genes up-regulated in heat stressed *mut-16* mutants exhibit increased chromatin accessibility, suggesting the observed changes in expression upon heat stress are due to *mut-16* mutants’ susceptibility to changes in chromatin status.

## DISCUSSION

Maintaining germ cell fate and totipotency is critical to preserving fertility. When genetic or environmental perturbations disrupt the delicate balance of gene expression in germ cells, these cells can be directly converted into other cell types and no longer be viable for fertilization and embryogenesis. In this study, we sought to understand how RNAi pathway mutants promote germ cell immortality. We utilized a mutant in the small RNA amplification machinery, *mut-16*, that, when cultured at elevated temperature from the L1 larval stage, is sterile. Through bioinformatic comparison of mRNA-seq libraries we identified genes whose expression is significantly differentially expressed in *mut-16* mutants cultured during heat stress. We further compared these data to genes that are differentially expressed in the *hrde-1* nuclear RNAi mutant, after it had been cultured for multiple generations at elevated temperature, ultimately reaching sterility. Strikingly, the up-regulated genes in both *mut-16* and *hrde-1* mutants at elevated temperature were predominantly spermatogenesis and soma-specific genes, and through additional experiments we conclude that somatic genes are being mis-expressed in the germ cells. Finally, using ATAC-seq libraries, we revealed that mis-expression of the soma-specific genes in germ cells correlates with increased chromatin accessibility in heat-stressed *mut-16* mutants. These findings reveal that a functioning RNAi pathway is essential in germ cells to prevent inappropriate expression of somatic genes in response to heat stress, and thus maintain cell fate and fertility.

The genes up-regulated in *mut-16* mutants experiencing heat stress are largely not known targets of the ALG-3/4, ERGO-1 or CSR-1 small RNA pathways. We also did not detect any change in small RNA expression targeting the majority of these genes, and very few other genes lose small RNAs in the *mut-16* mutant in a heat-specific manner. It is still plausible that there are more substantial changes in the small RNA pathway than we are detecting by small RNA-seq; rather than complete loss of certain small RNAs, there may be misrouting of small RNAs into incorrect Argonaute proteins differentially associated with gene silencing or anti-silencing pathways as has been observed in the absence of piRNAs or a cellular memory of piRNAs (81,82). We do see down-regulation of CSR-1 target genes in heat stressed *mut-16* mutants; however, we believe this is more likely due to the loss of the cellular identity of the germ cells. Because the up-regulated somatic genes are not known to be targets of any small RNA pathway nor can we detect changes in the small RNAs targeting these genes, we cannot explain their increased expression by changes in small RNAs directly targeting them.

We additionally observed aberrant expression of spermatogenesis genes in wild-type animals experiencing heat stress, *mut-16* mutants at permissive temperature, and even more dramatically in *mut-16* mutants experiencing heat stress. This aberrant expression may be due to a failure in turning off spermatogenesis gene expression at the spermatogenesis-to-oogenesis transition in heat-stressed and *mut-16* mutant animals. Like *mut-16* mutants, mutants in the *rsd-2* gene show increased expression of spermatogenesis genes (40). Additionally, mutants for some chromatin factors display similar transgenerational sterility phenotypes and accumulation of activating chromatin marks across many genes, yet increased expression is limited to the spermatogenesis genes (51,83). These data, along with our ATAC-seq data showing a global increase in chromatin accessibility at elevated temperature, would suggest that the spermatogenesis genes are inherently more susceptible to ectopic expression in an altered chromatin environment, whereas other genes may have additional layers of regulation. Furthermore, our data suggests that increased expression of spermatogenesis genes during oogenesis is unlikely to be the sole cause of heat-induced sterility, because wild-type animals experiencing heat stress and *mut-16* mutants at permissive temperature are fertile, albeit with both strains exhibiting moderately reduced brood sizes. Mating of heat-stressed *mut-16* hermaphrodites with males cultured at permissive temperature rescues fertility in the first generation at elevated temperature, implying that despite the increased expression of spermatogenesis genes the oocytes in *mut-16* can still be fertilized and produce viable offspring. However, after three subsequent generations at elevated temperature, *mut-16* mutant hermaphrodites have morphologically defective germlines and are sterile despite mating with wild-type males. This result reveals that heat-stressed *mut-16* mutants manifest a maternal effect that depends on the grandmaternal and maternal genotype and environmental conditions. It should be noted that our mRNA-seq and ATAC-seq were performed on animals cultured at elevated temperature for a single generation, but not on the second or third generation at elevated temperature. Thus, the changes in expression and chromatin accessibility we see in the first generation of heat stressed *mut-16* mutants may be further exacerbated in second generation heat-stressed *mut-16* mutants, which have reduced brood sizes upon mating, and the sterile third generation. Together, these data suggest that the sterility is affected by the inheritance of a transgenerational epigenetic signal.

Another potential cause of the sterility of *mut-16* mutants and the transgenerational sterility observed in *hrde-1* mutants could be massive up-regulation of transposable elements and repetitive regions of the genome. In the case of *hrde-1*, this accumulation of DNA damage over multiple generations could ultimately triggering sterility. However, in this work we demonstrate that there is no significant change in transposon mRNA expression between *mut-16* mutants at permissive and elevated temperature, and up-regulation of other repetitive elements is only modest. A similar result was observed by a recent study that looked at the expression of TE mRNAs in heat-stressed *prg-1* and *mut-16* mutants (80). Furthermore, multiple labs have shown that transgenerational sterility in multiple mutants, including *hrde-1*, can be suppressed by adding back a copy of the missing gene or returning the strain to permissive temperature, suggesting that the accumulation of DNA damage due to the failure to repress transposons and repetitive regions is unlikely to be the cause of the sterility (42,43,49).

We demonstrated here that P granule integrity is disrupted in *mut-16* mutants experiencing heat stress, however the changes did not correlate with a reduction in transcription of P granule components. It has been observed that some traditionally defined soma-specific genes and their transcriptional regulators may be transcribed at low levels within the germline, and thus disruption of P granules, which may serve as a repository for these maternally-derived, translationally-repressed mRNAs, allows germ cells to begin differentiation into neurons, muscle, and intestinal cells (1,4). Because small RNA pathways and P granules are intimately connected, it is difficult to determine whether disruption of P granules precedes the expression changes of soma-specific genes in the germline of small RNA amplification pathway mutants, or vice versa. Thus, two models could result in the germ cell to soma conversion observed in heat-stressed RNAi mutants: 1) P granules are disrupted in heat-stressed *mut-16* mutants, resulting in translation of soma-specific transcription factors, ultimately resulting in the increased expression of soma-specific genes or 2) Changes in the chromatin status of soma-specific gene loci occur due to the inability of heat-stressed *mut-16* pathway mutants to maintain appropriate chromatin marks at these loci, ultimately resulting in the disruption of P granules, which further contributes to the observed germ cell to soma conversion.

Our data supports the second model, however the two models are by no means mutually exclusive. First, P granule depletion does not result in any detectible change in the expression of somatic genes in germ cells in day one adults (10), which we do observe in heat-stressed RNAi pathway mutants. It was, however, shown that older P granule-depleted adults do exhibit up-regulation of somatic transcripts (11), suggesting that, in *mut-16* mutants, changes in transcript levels of soma-specific genes in the germline are occurring earlier than in P granule mutants. Thus, while P granules clearly play a fundamental role in maintaining the cellular identity of germ cells, their disruption is not likely to be the sole mechanism by which germ cell to soma conversion occurs. Therefore, we propose that the increase in expression of somatic genes in RNAi pathway mutants experiencing heat stress may be directly tied to global changes in chromatin modification based on the following lines of evidence: 1) RNAi pathway mutants have fertility defects similar to those of chromatin modifying enzyme mutants such as the H3K4 methyltransferase *set-2*, the H3K9 methyltransferase *met-2* or the H3K4me2 demethylase *spr-5* (45–51); 2) chromatin modifying enzymes act downstream of the RNAi pathway and through the nuclear RNAi pathway (12,16,42,84–90); 3) chromatin modifying enzymes such as the H3K4 methyltransferase SET-2 acts to maintain repression of somatic genes in germ cells, thus preventing premature germ cell differentiation (2,6–8); and 4) RNAi pathway mutants have global increases in chromatin accessibility along gene bodies in isolated germline nuclei of heat-stressed wild-type animals and, more severely, in heat-stressed *mut-16* mutants, suggesting a loss of chromatin compaction at these loci (Figure 8).

It is interesting to note that despite a global change in chromatin accessibility in heat stressed animals, and even more so in heat stressed *mut-16* mutants, only a subset of genes exhibit differential expression in *mut-16* mutants at elevated temperatures. These genes are perhaps more susceptible to changes in their chromatin status or lack additional layers of transcriptional regulation. Thus, the RNAi machinery, including *mut-16* and the small amplification complex, and the nuclear RNAi pathway, play a key role in the regulation of germ cell immortality, likely by maintaining a proper epigenetic state. Furthermore, recent studies in *Drosophila melanogaster* have shown that germ cell-specific loss of each of the H3K9me3 pathway components SetDB1, Wde, HP1a, and Su(Var)2–10, which links the nuclear piRNA-targeting machinery to the chromatin silencing machinery in *D. melanogaster* ovaries, results in ectopic expression of spermatogenesis and somatic genes within the female germline, ultimately leading to the loss of female germ cell identity (91–93). These findings suggest that the susceptibility of germ cell identity to changes in the chromatin status, in combination with perturbations in small RNA pathways, may be an evolutionarily conserved mechanism. In conclusion, our results uncover a new role for the small RNA pathway in maintaining germ cell fate during heat stress. Through its interaction with chromatin modifying pathways, the small RNA amplification machinery ensures proper gene expression across generations and restricts somatic expression to maintain cellular identity and fertility.

## DATA AVAILABILITY

High-throughput sequencing data for mRNA-seq, small RNA-seq, and ATAC-seq experiments generated during this study are available through Gene Expression Omnibus (GEO #: GSE134573). De-multiplexed raw sequencing data, in fastq format, for N2 and *hrde-1* mRNA-seq libraries used from (42) were obtained from NCBI’s Gene Expression Omnibus (GEO #: GSE74405).

## Supplementary Material

gkaa174_Supplemental_File
