# Supplementary material for: RNAi pathways repress reprogramming of C. elegans germ cells during heat stress
Source: Nucleic Acids Res. 2020 Mar 18;48(8):4256–73. doi: 10.1093/nar/gkaa174 (PMC7192617; doi:10.1093/nar/gkaa174)
Supplement: gkaa174_Supplemental_File [file gkaa174_supplemental_file.pdf]

Supplemental figures and figure legends

Supplemental Figure 1

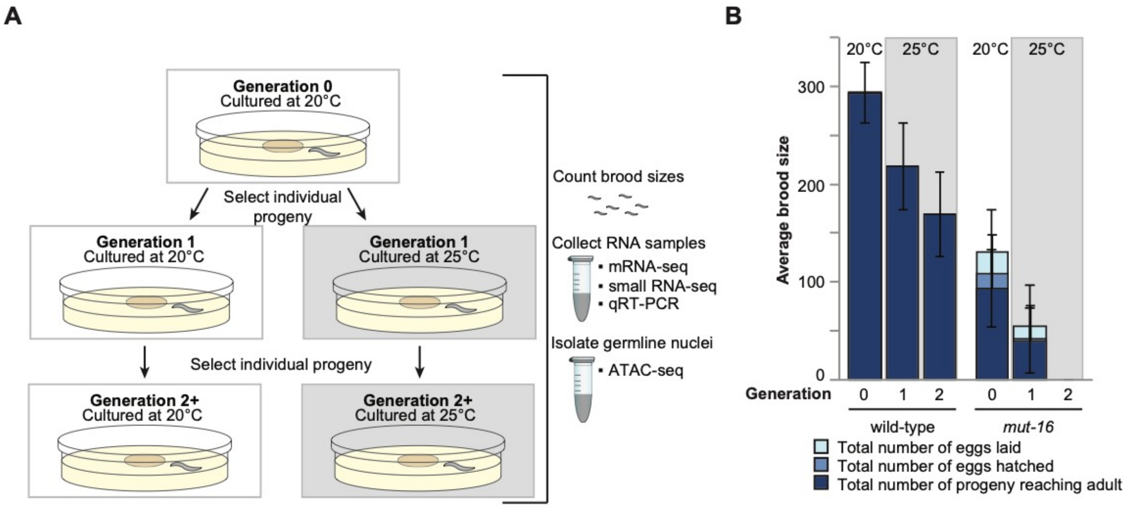

Supplemental Figure 1. (A) Schematic for temperature-shift assays. For brood size assay, synchronized L3s were shifted to 25°C, and for all remaining experiments, synchronized L1s were shifted to 25°C. **(B)** *mut-16* mutants have reduced brood sizes. Over-laid bar graph indicates total number of eggs laid (light blue), total number of hatched larvae (medium blue), and total number of animals that survive to adulthood (dark blue). For each generation, n=30 broods. Error bars indicate standard deviation.

Supplemental Figure 2

A

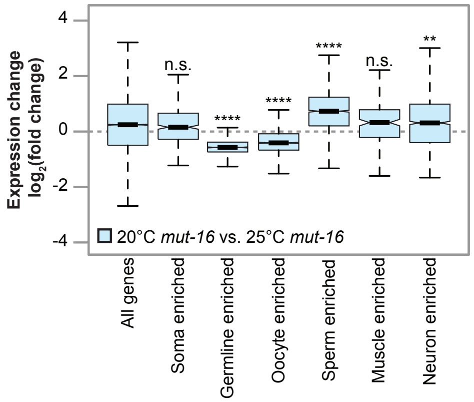

B

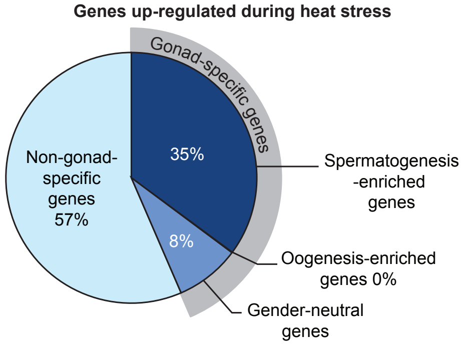

Enrichment analysis of genes up-regulated during heat stress

Gene list	log_2(fold change)	Significance
**Spermatogenesis-enriched genes**	2.56	****
**Oogenesis-enriched genes**	-5.80	****
**Gender-neutral gonad genes**	-0.71	****

C

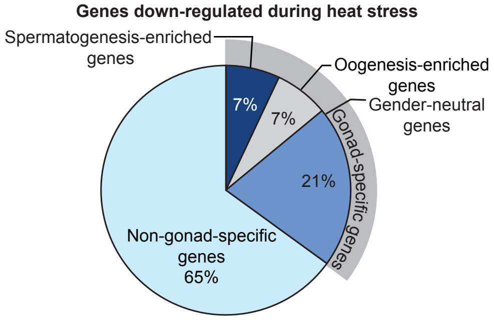

Enrichment analysis of genes down-regulated during heat stress

Gene list	log_2(fold change)	Significance
**Spermatogenesis-enriched genes**	0.32	n.s.
**Oogenesis-enriched genes**	0.81	n.s.
**Gender-neutral gonad genes**	0.61	*

Supplemental Figure 2. (A) Comparison of expression changes in *mut-16* mutants cultured at 25°C compared to *mut-16* mutants cultured at 20°C for published enriched gene sets. Notches indicate the 95% confidence interval of the median; black line indicates median. **(B-C)** Percentages of gonad-specific and non-gonad-specific genes represented in the genes up-regulated (B) and down-regulated (C) in wild-type and *mut-16* mutants at 25°C compared to wild-type and *mut-16* mutants at 20°C. Enrichment analysis for spermatogenesis, oogenesis, and gender-neutral genes amongst the genes up-regulated (B) and down-regulated (C) during heat stress is shown in the table below. n.s. denotes not significant and indicates a p-value > 0.05, * indicates a p-value ≤ 0.05, ** indicates a p-value ≤ 0.01, and **** indicates a p-value ≤ 0.0001.

Supplemental Figure 3

A

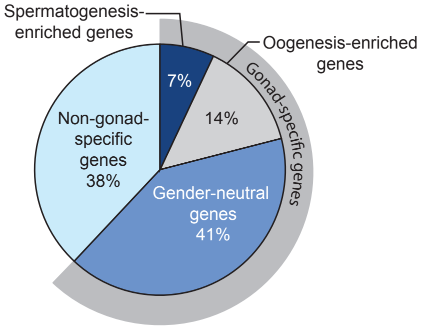

Enrichment analysis of genes down-regulated in *mut-16* mutants at 25°C

Gene list	log_2(fold change)	Significance
**Spermatogenesis-enriched genes**	0.26	n.s.
**Oogenesis-enriched genes**	1.90	****
**Gender-neutral gonad genes**	1.60	****

B

Enrichment analysis of small RNA targets for genes down-regulated in *mut-16* mutants at 25°C

Gene list	log_2(fold change)	Significance
**ALG-3/4 targets**	0.65	*
**ERGO-1 targets**	-0.43	n.s.
***mutator* targets**	-0.58	n.s.
**piRNA targets**	-0.01	n.s.
**CSR-1 targets**	2.13	****

Supplemental Figure 3. (A) Percentages of gonad-specific and non-gonad-specific genes represented in the genes down-regulated specifically in *mut-16* mutants at 25°C, compared to wild-type animals at 20°C and 25°C and *mut-16* mutants at 20°C. Enrichment analysis for spermatogenesis, oogenesis, and gender-neutral genes amongst the genes down-regulated in *mut-16* mutants at 25°C is shown in the table below. **(B)** Enrichment analysis for small RNA pathway target genes represented in the genes down-regulated exclusively in *mut-16* mutants at 25°C. n.s. denotes not significant and indicates a p-value > 0.05, * indicates a p-value ≤ 0.05, **** indicates a p-value ≤ 0.0001.

Supplemental Figure 4

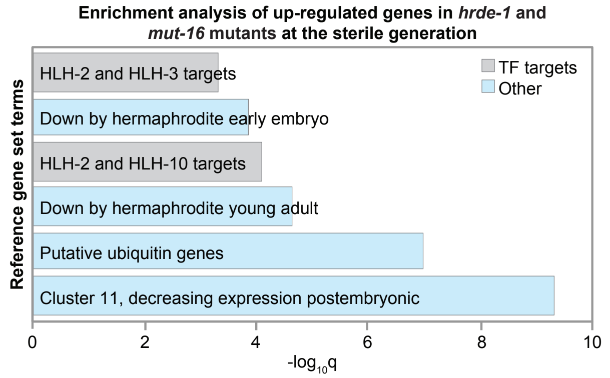

Supplemental Figure 4. Gene enrichment analysis of genes up-regulated in both *mut-16* and *hrde-1* mutants at the sterile generation after culturing at elevated temperature using gene reference lists (TF targets (gray) and Other (light blue)) from WormExp ($\log_{10}Q \geq 2$ and FDR < 0.05).

Supplemental Figure 5

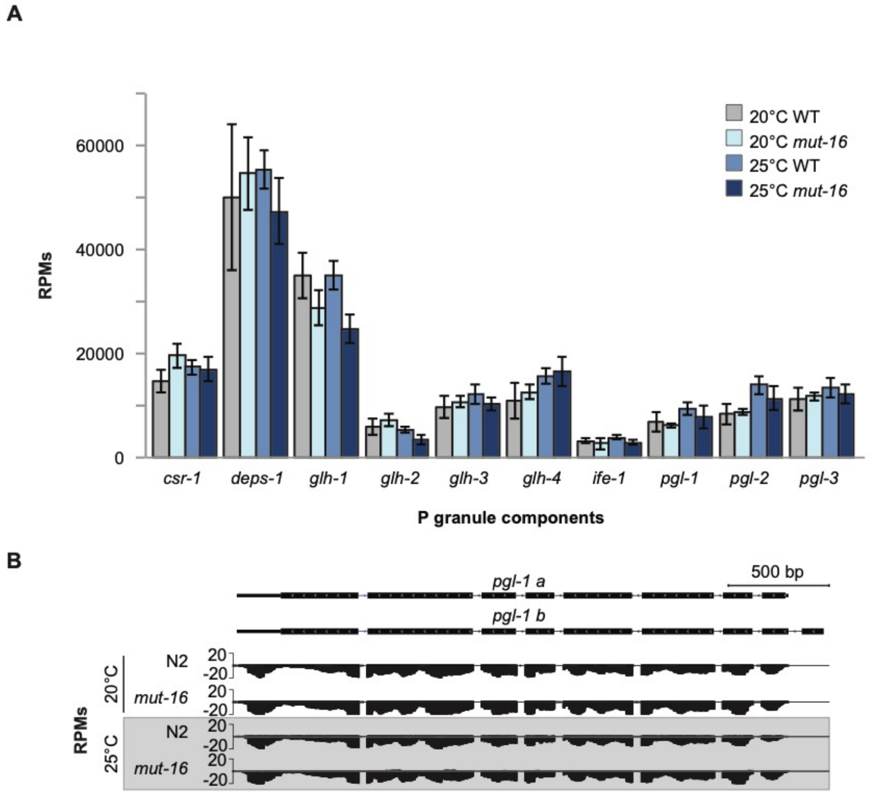

Supplemental Figure 5. (A) mRNAs mapping to P granule components are counted, in reads per million (RPMs), from wild-type and *mut-16* mutants cultured at 20°C and 25°C. Error bars indicate standard deviation between replicate libraries. **(B)** mRNA-seq reads, in reads per million (RPMs) are mapped to the genomic locus of PGL-1 in wild-type and *mut-16* mutants cultured at 20°C and 25°C.

Supplemental Figure 6

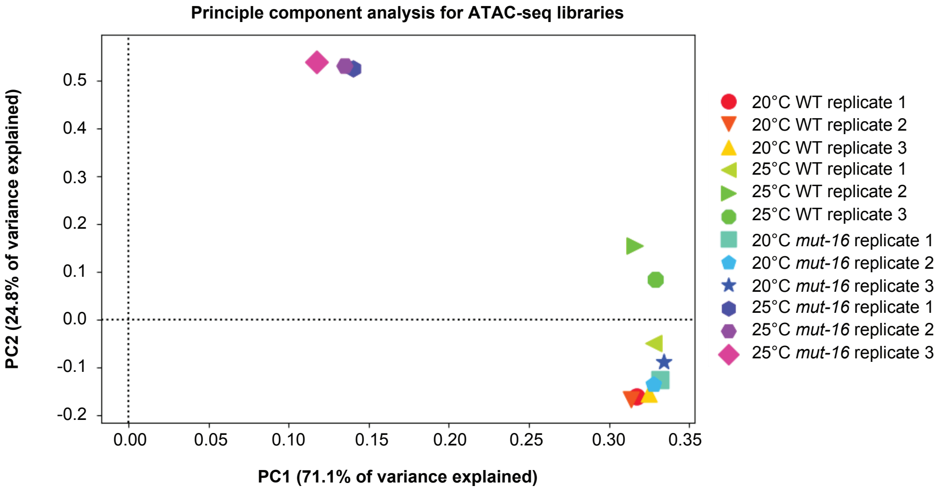

Supplemental Figure 6. Principle component analysis for ATAC-seq libraries. Libraries are in biological triplicates.

Supplemental Tables.

Supplemental Table 1. Library mapping statistics.

Library	Total number of reads	Reads mapping to WS258 genome	Reads mapping to WS258 mRNA, ncRNA, and pseudogenic transcripts	Reads mapping to WS258 transposon transcripts
N2_20C_1_mRNA	40,478,887	36,964,023	19,960,805	17,400
N2_20C_2_mRNA	37,712,342	34,452,658	18,569,724	20,056
N2_20C_3_mRNA	39,768,225	35,889,709	24,219,091	18,910
N2_25C_1_mRNA	39,785,156	36,887,658	21,675,767	20,083
N2_25C_2_mRNA	44,791,266	40,811,177	28,472,933	24,369
N2_25C_3_mRNA	43,083,005	39,945,170	26,746,924	22,852
*mut-16*_20C_1_mRNA	39,141,147	35,666,080	20,894,813	32,121
*mut-16*_20C_2_mRNA	43,248,000	39,308,071	21,391,843	27,384
*mut-16*_20C_3_mRNA	54,956,698	50,063,712	25,965,885	29,961
*mut-16*_25C_1_mRNA	24,685,397	22,549,327	15,432,009	19,251
*mut-16*_25C_2_mRNA	44,004,746	39,635,799	31,774,073	33,826
*mut-16*_25C_3_mRNA	46,767,699	42,560,583	34,195,602	36,042
N2_20C_1_small_RNA	15,234,469	11,795,411	9,964,465	466,967
N2_20C_2_small_RNA	16,942,967	13,458,071	11,401,866	568,202
N2_20C_3_small_RNA	14,561,315	11,350,459	9,579,543	480,050
N2_25C_1_small_RNA	28,323,386	23,084,410	19,060,841	1,109,291
N2_25C_2_small_RNA	16,662,554	13,311,365	11,012,073	646,791
N2_25C_3_small_RNA	13,253,801	5,580,040	4,760,502	266,391
*mut-16*_20C_1_ small_RNA	27,965,271	21,415,928	17,925,273	345,833
*mut-16*_20C_2_ small_RNA	16,407,955	12,409,115	10,069,275	210,135
*mut-16*_20C_3_ small_RNA	15,640,099	12,089,120	10,076,941	184,814
*mut-16*_25C_1_ small_RNA	16,355,902	11,538,825	7,908,720	178,692
*mut-16*_25C_2_ small_RNA	16,508,845	13,361,956	9,140,188	222,504
*mut-16*_25C_3_ small_RNA	14,847,509	12,290,524	8,842,206	157,760
N2_20C_1_ATAC	27,010,582	26,617,066	--	--
N2_20C_2_ ATAC	30,139,182	29,951,867	--	--
N2_20C_3_ ATAC	39,746,363	39,288,387	--	--
N2_25C_1_ ATAC	29,736, 773	29,274,431	--	--
N2_25C_2_ ATAC	32,752,234	31,448,960	--	--
N2_25C_3_ ATAC	30,680,231	29,884,443	--	--
*mut-16*_20C_1_ ATAC	27,191,206	26,830,974	--	--
*mut-16*_20C_2_ ATAC	25,143,065	24,869,785	--	--
*mut-16*_20C_3_ ATAC	27,642,635	26,898,506	--	--
*mut-16*_25C_1_ ATAC	27,001,433	24,667,643	--	--
*mut-16*_25C_2_ ATAC	22,511,278	18,728,422	--	--
*mut-16*_25C_3_ ATAC	26,612,955	25,225,745	--	--

Supplemental Table 2. Oligonucleotide sequences.

Primer name	Sequence
CP625_rpl-32_F	CAAGGTCGTCAAGAAGAAGC
CP625_rpl-32_R	GGCTACACGACGGTATCTGT
CP1416_pgl-1_F	TGTTGAGCTCACGGAACTTG
CP1417_pgl-1_R	GATCGGCAGGTTCAGATTTC
CP1418_glp-1_F	CTCAAAATGAATGCGCAGAA
CP1419_glp-1_R	TATCCCGAGTCGCATACACA
CP1420_cdc-42_F	CTGCTGGACAGGAAGATTACG
CP1421_cdc-42_R	CTCGGACATTCTCGAATGAAG
CP1424_rab-3_F	GCCTTCGTCTCTACTGTCGG
CP1425_rab-3_R	CGGCGGTATCCCAGATTTGA
CP1426_myo-3_F	GCCTACGCTGATGCTCAGAA
CP1427_myo-3_R	CCTTCTGGCGTTGTTCCTCT
CP1430_faah-1_F	TTCACACCAACACCTGCACT
CP1431_faah-1_R	TGGAATGACTGTATGTCCGGC
CP1432_F57F4.1_F	GGCCGCATCAATTTCAGACC
CP1433_F57F4.1_R	CGTGTTAACTCGGGCCTCTT
CP1434_C15H11.1_F	GGGAGGGCAAGAGACTGTTC
CP1435_C15H11.1_R	CCCAAGCTCCCTCACAAGTA
CP1436_clp-8_F	AGATGACGCATTTCCGGCTA
CP1437_clp-8_R	TCGTCAACCGCCGAAATACA
CP1438_ssp-35_F	TCGTCTCGGAGTCGGATTCT
CP1439_ssp-35_R	GACGAACACGGTAGTGCTCA

Supplemental Table 3. Average technical replicate Ct values for qRT-PCR biological replicates.

Sample	20°C Wild-type (N2)		25°C Wild-type (N2)		20°C *mut-16*		25°C *mut-16*	
**Replicate**	**1**	**2**	**1**	**2**	**1**	**2**	**1**	**2**
**qRT-PCR for spermatogenesis genes using hermaphrodite RNA**								
rpl-32	20.19	19.16	20.02	19.61	20.79	19.90	18.95	20.76
**F57F4.11**	33.15	28.72	31.13	31.72	33.82	27.07	26.82	27.14
**C15H11.1**	27.20	25.16	25.99	27.53	24.37	22.65	21.53	23.41
clp-8	32.59	30.05	30.15	32.84	24.82	25.43	23.12	25.33
**qRT-PCR for spermatogenesis genes using gonad tissue RNA**								
cdc-42	21.35	21.84	24.07	23.77	20.66	23.22	20.18	22.03
**F57F4.11**	33.05	32.10	34.33	34.15	30.33	32.60	29.33	31.03
**C15H11.1**	30.48	31.39	32.51	33.13	28.99	31.60	28.17	29.31
clp-8	37.09	36.09	38.19	38.27	32.38	36.19	32.20	34.3
ssp-35	30.32	30.43	32.35	31.99	27.87	30.42	28.05	28.31
**qRT-PCR for soma-specific genes using gonad tissue RNA**								
cdc-42	24.96	23.06	28.40	21.55	21.80	25.77	28.34	24.20
rab-3	29.55	31.10	30.60	27.43	31.17	29.76	26.36	22.16
myo-3	26.24	27.96	30.60	26.26	29.33	28.13	24.42	22.13
faah-1	25.44	27.12	29.91	25.49	29.47	27.11	24.06	21.28
**qRT-PCR for germline-specific genes using gonad tissue RNA**								
cdc-42	23.33	23.31	24.57	25.30	27.28	23.21	24.33	24.96
pgl-1	21.57	21.11	23.08	23.25	24.76	21.15	21.62	22.85
